# Assessment of Colorectal Cancer Risk Factors through the Application of Network-Based Approaches in a Racially Diverse Cohort of Colon Organoid Stem Cells

**DOI:** 10.3390/cancers15143550

**Published:** 2023-07-09

**Authors:** Matthew Devall, Stephen Eaton, Cynthia Yoshida, Steven M. Powell, Graham Casey, Li Li

**Affiliations:** 1 Department of Family Medicine, University of Virginia, Charlottesville, VA 22903, USAll8nv@virginia.edu (L.L.); 2 Digestive Health Center, University of Virginia, Charlottesville, VA 22903, USA; 3 Center for Public Health Genomics, University of Virginia, Charlottesville, VA 22908, USA; gc8r@virginia.edu; 4 Department of Public Health Sciences, University of Virginia, Charlottesville, VA 22908, USA; 5 University of Virginia Comprehensive Cancer Center, University of Virginia, Charlottesville, VA 22908, USA

**Keywords:** colon organoid, stem cell, racial disparities, colorectal cancer risk, network analysis, WGCNA, smoking, aging, body mass index

## Abstract

**Simple Summary:**

Risk factors for colorectal cancer (CRC) include age, body mass index, race, smoking history, and sex. However, molecular mechanisms underlying these associations remain unclear. To better define transcriptional mechanisms impacting risk, we hypothesized that stem-cell-enriched colon organoids derived from the healthy epithelium of a diverse, average-risk patient population would provide insight into genes associated with these risk factors. We used network-based approaches to better define transcriptional mechanisms involved in CRC risk and incorporated external, publicly available datasets to prioritize likely biological drivers.

**Abstract:**

Numerous demographic factors have been associated with colorectal cancer (CRC) risk. To better define biological mechanisms underlying these associations, we performed RNA sequencing of stem-cell-enriched organoids derived from the healthy colons of seven European Americans and eight African Americans. A weighted gene co-expression network analysis was performed following RNA sequencing. Module–trait relationships were determined through the association testing of each module and five CRC risk factors (age, body mass index, sex, smoking history, and race). Only modules that displayed a significantly positive correlation for gene significance and module membership were considered for further investigation. In total, 16 modules were associated with known CRC risk factors (*p* < 0.05). To contextualize the role of risk modules in CRC, publicly available RNA-sequencing data from TCGA-COAD were downloaded and re-analyzed. Differentially expressed genes identified between tumors and matched normal-adjacent tissue were overlaid across each module. Loci derived from CRC genome-wide association studies were additionally overlaid across modules to identify robust putative targets of risk. Among them, *MYBL2* and *RXRA* represented strong plausible drivers through which cigarette smoking and BMI potentially modulated CRC risk, respectively. In summary, our findings highlight the potential of the colon organoid system in identifying novel CRC risk mechanisms in an ancestrally diverse and cellularly relevant population.

## 1. Introduction

Colorectal cancer (CRC) represents the third most common cancer in men and women, as well as the third leading cause of cancer-related death [1]. A range of lifestyle and demographic factors have been associated with increased risk. Interestingly, the strengths of these associations have been shown to be affected by colon-cancer-sidedness. Aging is considered the strongest risk factor for CRC, and a left-to-right shift in colon cancer incidence is observed with increased age [2]. Similarly, females are more likely to develop right-side colon cancer, as too are African American (AA) males [3]. Indeed, AAs are approximately 30% more likely to develop right- than left-side CRC [4]. Large case-control studies also reported stronger associations with smoking in proximal (right) than distal (left) colon cancers [5], while a systematic review of prospective studies that included data from approximately nine million participants revealed strong, positive associations for body mass index (BMI) and cancer of both the right and left colon [6]. The biological mechanisms underpinning the associations between lifestyle and demographic variation in CRC risk are still unclear. However, growing evidence suggests that side-specificity should be an important consideration when designing such studies.

Traditionally, mechanistic studies of CRC have been performed in mouse models or cancer cell lines derived from CRC tumors. However, these models are not without limitations. Species-specific patterns of CRC tumorigenesis are attributed to various mouse models [7]. Further, while some mouse models are designed to mimic left-side colon cancers [8], the mouse colon is smooth and not divided into sections, absent of taenia coli, and not compartmentalized by haustra [9]. The presence of activated oncogenic signaling and somatic mutation burdens in CRC cell lines raises important questions about their use in evaluating mechanisms associated with risk. Cancer initiation occurs in healthy, non-cancerous cellular environments. Further, few AA CRC lines have been developed [10], and no study monitoring the effects of racial disparities can be performed in mouse models. Thus, while there is a pressing need to define mechanisms contributing to CRC risk, the limitations of traditional models make this challenging.

The development of the colon organoid model [11] presents an important option for the study of healthy human colon epithelial cells and CRC initiation. These adaptable, patient-derived models recapitulate the epithelial stem cell niche environment of the colon crypt from which they are derived and can be generated from any region of the colorectum. As such, organoids provide an opportunity to study the impact of risk factors across a diverse, average-risk patient population. However, it is important to note that traditional organoid culture results in a heterogenous mixture of cells of the stem cell niche. A previous work showed that organoids could be enriched for a homogenous pool of multipotent Lgr5^+^ stem cells through the addition of valproic acid and CHIR99021 [12]. Stem cells play a crucial role in normal crypt biology, maintaining homeostasis through the complete repopulation of the epithelial monolayer every 5–7 days [13]. Resident stem cells also represent one population from which colorectal adenomas may be derived [14,15,16]. The profiling of this specific population, therefore, offers the potential to identify genes associated with CRC risk factors.

A common approach for the study of specific disease conditions is the assessment of individual gene expression differences derived from omics-based approaches, such as RNA-seq. However, genes do not act in isolation and frequently do not serve a solitary function. Indeed, previous research has shown that individual genes may be represented in an average of 75.3 gene ontology (GO) terms [17,18]. Methods such as weighted gene co-expression analysis (WGCNA) provide important advantages over single-gene analysis by considering how groups of highly co-expressed genes (modules) jointly affect complex diseases [19,20]. The use of WGCNA and other network-based approaches has led to a better understanding of cell state and dynamics by highlighting the extensively complex systems through which gene–gene interactions occur [21]. Given the cellular specificity of such interactions, averaging signal across highly heterogeneous mixtures can diminish mechanistic inference [22]. However, when considered in cell populations relevant to the outcome of interest, such methods have the power to better define disease-relevant transcriptional machinery [23]. Our group has previously accounted for differences in cell composition in our colon organoid model to assess how various environmental exposures can drive CRC-related transcriptomic signals, providing mechanistic insight into the role of the environment in the modulation of disease risk [22,24,25,26]. Here, we develop a framework for the analysis of environmental exposure risk in the colon organoid system that centers on the ability of WGCNA to identify robust, biologically relevant modules of gene co-expression and their likely CRC-related drivers [24,26]. However, this framework has yet to be considered to determine the effects of patient-specific lifestyle and demographic factors on CRC risk in stem cells of healthy colon organoids.

We generate a genetically diverse, biracial set of normal, right-colon organoids (*n* = 15, 8 AA) and grow them in media designed to enrich stem cell populations [12]. We hypothesize that, through the use of WGCNA [20], we can identify modules of gene co-expression related to five well-established CRC risk factors: age, BMI, sex, smoking history, and ancestry (AA vs. European American (EA)), and that relating genes within these modules to publicly available CRC data can provide mechanistic insight into the effects of lifestyle and demographic factors on CRC risk. By overlaying data from a publicly available CRC cohort, we identify novel drivers of environmental CRC risk. Our results highlight the power of coupling an organoid system with network-based approaches to dissect CRC risk mechanisms.

## 2. Materials and Methods

### 2.1. Patient Selection

Subjects (*n* = 15) undergoing standard-of-care colonoscopy procedures were recruited for a biopsy study at the University of Virginia (UVA). All participants provided written informed consent prior to participating in the research study, and all research procedures were approved by the Institutional Review Board of the UVA (HSR #19439).

### 2.2. Biopsy Collection and Establishment of Colon Organoids in Matrigel

Normal human colon organoids were established using minimal modification of the method described by Sato et al. [11] and as previously described [25,26,27,28]. Four biopsies from right and four biopsies from left colons were taken at the time of colonoscopy using standard forceps. These biopsies were obtained from immediately distal to the hepatic flexure (right colon) or immediately distal to the splenic flexure (left colon). Biopsies were placed into DMEM/F12 medium with 10% fetal bovine serum (FBS) and 100 U/mL penicillin and 100 μg/mL streptomycin on ice. Biopsies were then immediately transported to the laboratory, and whole crypts were isolated using methods previously described [11,29]. Briefly, samples were washed 3× with DPBS at room temperature. An amount of 10 mL DPBS containing 9 mM EDTA was added to each colon biopsy and was then incubated for 20 min at room temperature. Samples were manually inverted every 2–3 min. To avoid crypt adhesion, all tubes, tips, and pipets were conditioned with wash medium (DMEM/F-12, 10% FBS, 10 mM HEPES, 2 mM L-glutamine, 1X Pen-Strep (100 U/mL penicillin and 100 μg/mL streptomycin), and 1× Glutamax). Following incubation, DPBS/EDTA was removed. An amount of 10 mL DPBS absent of EDTA was then added and mixed 8–10× via manual pipetting. After the tissue settled, supernatant was collected in a 15 mL tube. This process was repeated a total of three times. Collected supernatant was spun at 1200 rpm at 4 °C for five minutes. To not disturb the pellet, all but 2 mL of supernatant from each 15 mL tube was removed and combined in a clean 50 mL tube. Using a conditioned 5 mL pipet, the remaining 2 mL from each tube was then combined into one 15 mL tube and spun at 1200 rpm at 4 °C for five minutes. After this, the resulting supernatant was removed. An amount of 10 μL complex medium (45% wash medium, 50% L-WRN conditioned medium, 10 nM Gastrin, 10 μM Y27632, 1X B27 supplement, 1X N2 supplement, 1 mM n-Acetylcysteine, 50 ng/μL EGF, 10mM Nicotinamide, 500 nM A83, and 10 μM SB202190) was added and mixed by manual pipetting. An amount of 200 μL Matrigel was then added and mixed. A total of 35–50 μL of each sample was then plated at the center of wells of a 48-well plate and incubated at 37 °C for 15 min. An amount of 300–500 μL complex medium was then added, and samples were returned to the incubator. Organoids were checked and fed after 24 h before being passaged as needed (every 3–5 days) and frozen. For the purpose of this study, only organoids generated from the right colon were considered, given the well-recognized disparities that exist between AA and EA individuals in right-side colon cancer [30].

### 2.3. Stem Cell Enrichment of Colon Organoids

Each line was grown as described previously [24,25,26,27,28] using a split ratio of 1:3 for routine passaging. Organoids were split five days prior to stem cell enrichment and nine days prior to harvesting. Colon organoids were grown in complex medium for a period of five days, replenishing with fresh medium on day three. On day five, medium was aspirated, and 500 µL/well CV medium (10 mL complex medium, 10 µL 5 mM CHIR99021 (Stemcell Technologies: 72054, Vancouver, Canada), and 100 µL 100 mM valproic acid (Stemcell Technologies: 72292)) was added to each well, as previously described [12]. Following this, organoids were grown for four additional days.

### 2.4. RNA Isolation from Colon Organoids and RNA Processing

For RNA isolation, media under differing conditions were gently removed while being careful not to disturb the Matrigel. Next, 300 μL RNA RA1 lysis solution with 6 μL TCEP (Macherey-Nagel; 740902.50, Düren, Germany) was added to each well, and the lysate was collected in a 1.5 mL Eppendorf tube. Each tube was vortexed extensively and frozen at −80 °C until extraction. RNA extraction was carried out according to the manufacturer’s protocol of a NucleoSpin RNA Mini Kit (Macherey-Nagel; 740955.250, Oensingen, Switzerland) Poly-A selection and cDNA synthesis were performed using a TruSeq Stranded mRNA kit (Illumina; 20020595, San Diego, CA, USA). RNA-seq data were generated using NovaSeq at the Genomics Core Facility of the Icahn School of Medicine, Mount Sinai. Raw, paired Fastq files were generated following two sequencing runs. Fastq files from both runs were merged prior to quality control, read, and adaptor-trimmed using Trim Galore! [31]. Trimmed Fastq pairs were then aligned to release 104 of the Ensembl human reference genome [32] using STAR aligner in two-pass mode under default settings [33]. Expected gene counts and transcripts per million (TPM) were calculated from bam files using the RSEM algorithm [34].

### 2.5. Weighted Gene Co-Expression Network Analysis (WGCNA)

To mitigate the potential effects of spurious correlations among lowly expressed genes, genes with fewer than 10 normalized counts in 10 samples were removed using custom code. The purpose of this consideration was to safeguard gene–gene correlations from spurious findings driven by the relatively large degree of standard deviation observed among lowly expressed genes. A total of 16,757 genes remained post-filtering across 15 samples. A blinded variance-stabilizing transformation was applied to raw counts [35] prior to WGCNA [20]. A WGCNA was performed across all samples using the default argument of each function [20] with a few notable exceptions: the network was raised to a soft-thresholding power of seven; signed hybrid parameters were specified throughout; module size was set to 30; a deep split of three was used; and resulting modules with correlations greater than 0.8 were merged. Only modules where gene significance (GS) and module membership (MM) were significantly correlated were considered for further investigation. A pathway enrichment analysis was performed by uploading module members to STRING [36] using an FDR stringency set at 5% and setting the whole genome as a background. Modules of interest were plotted in Cytoscape [37] following exportation from an inbuilt function of the WGCNA.

### 2.6. Mapping Genes to CRC GWAS Loci

CRC GWAS index SNPs were downloaded from the GWAS catalog, as described by Huyghe et al. and Fernandez-Rozadilla et al. [38,39,40]. Genes with at least a single nucleotide of one exon overlapping a 1MB interval centered on an index SNP were included in the analysis. The genomic location of SNPs was based on their hg38 coordinates. BiomaRt [41,42] was used to determine GrCH38 gene coordinates of nearby genes.

### 2.7. Analysis of Publicly Available Data

To relate findings observed in stem-cell-enriched colon organoids with CRC-related events, we downloaded data from the TCGA-COAD dataset (*n* = 41) [43] using TCGAbiolinks [44]. A repeated measures regression model was fitted to analyze differences in gene expression between CRC tumors and matched normal-adjacent tissue (NAT) using DESeq2 while specifying a log_2_fold threshold of 0.5 [35].

### 2.8. Single-Cell Deconvolution of Bulk RNA-seq Data

To validate the enrichment of stem cell profiles within our dataset, we downloaded scRNA-seq data from a large, previously published colon biopsy dataset [45]. As previously described [24,25,26], we generated transcripts per million (TPM) mapped reads for each sample in bulk and singe-cell data. A signature matrix was generated using the following settings: minimum expression = 0.1, minimum number of genes = 20, and q-value = 0.001. For cell scoring, absolute mode and S-batch were both specified. A total of 500 permutations were used to determine cell score. Deconvolution performance was assessed using CIBERSORTx [46]. All samples were found to have significant empirical *p*-values for tests comparing imputed cell score to random chance mixtures. To define differences in stem cell content between samples grown in routine, complex media conditions [11,24,47,48] and those grown here in CV media [12], we performed a mixed-effects regression analysis on absolute stem cell score while accounting for sample pairing [49].

### 2.9. Quantitative PCR (qPCR) of Stem Cell Profiles

Gene expression validation experiments were performed using RNA isolated from six matched colon organoid lines grown in CV and complex media. Lines were chosen based upon remaining quantities of RNA following RNA sequencing. RNA for qPCR was isolated as described above. RNA concentration was determined with a Qubit fluorometer (Thermo-Fisher, Waltham, MA, USA). A minimum of 500 ng of total RNA was reverse-transcribed to first-strand cDNA using a High-Capacity cDNA Reverse Transcription Kit (Thermo-Fisher, 4368814). First-Strand cDNA was used for Taq-Man qPCR monitored with a QuantStudio Real-Time PCR analyzer (Thermo Fisher). Pre-Designed TaqMan Gene Expression Assays (Thermo Fisher) were used for quantification of several genes. Beta glucuronidase (GUSB) was used as a control gene to determine delta-CT values. These values were then used as input for a linear regression analysis while accounting for sample pairing. Known markers of stem cells (*LGR5* and *MEX3A*) and proliferation (*MKI67*) were tested for differences in expression between CV and complex media.

## 3. Results

### 3.1. Generation of Colon Organoid Dataset

A total of 15 independent colon organoid lines were generated from normal right-colon biopsies at the time of routine colonoscopy and grown in CV medium to enrich their stem cell populations (Table 1). On average, 53.49 million uniquely mapped reads were generated for each sample, with an average mapping efficiency of 85.55% (Table S1). Selected lines were balanced for ancestry (AA = 8, EA = 7) across a broad age (22–69; median = 54). The expected increase in stem cell content in the CV medium was confirmed using single-cell deconvolution [46] and publicly available scRNA-seq data [45] (Figures S1 and S2). A regression analysis of matched samples grown in standard, complex medium and CV conditions revealed a significant increase in stem cell content for CV (*p* = 1.26 × 10^6^). Stem cells grown in CV media accounted for 41.04% of the total cellular content, representing a 28.14% (3.18-fold) increase over the total content grown under normal conditions. We performed qPCR in six CV and six matched lines grown in standard complex medium [11,47]. We identified significant increases in the expressions of two known colon stem cell markers, LGR5 (*p* = 2.00 × 10^4^) and MEX3A (*p* = 1.88 × 10^5^) [50], as well as the proliferation marker MKI67 (*p* = 2.77 × 10^3^) (Figure S3). These findings are in line with those expected from a slowly dividing MEX3A+/LGR5+ stem cell pool [50].

### 3.2. WGCNA of Stem-Cell-Enriched Colon Organoids

To define transcriptional networks of colon stem cells, we performed a WGCNA [20] to first identify modules of co-expression and then determine the extent of their significant associations with known CRC-related factors (Figure S4A–D). Our network consisted of 56 modules of co-expression. Modules were then related to five CRC risk factors: ancestry (AA vs. EA), biological sex, age (continuous trait), BMI (continuous trait), and smoking status (never versus ever a smoker). A total of 23 significant associations (*p* < 0.05) were identified between modules and traits of interest. GS and MM were calculated for each gene and significant module. Modules that displayed a significant, positive correlation for GS and MM were considered for downstream analysis, with two being excluded. In total, 19 of 21 modules were unique to one trait and survived quality control measures (Table 2). To identify biologically relevant drivers of CRC risk, genes identified within significant modules were cross-referenced with differentially expressed genes (DEGs) identified in a re-analysis of a tumor versus matched NAT from TCGA-COAD (Table S2). To further prioritize drivers of likely disease risk mechanism, we considered a second layer of supporting evidence by determining which module members were also known to be gene targets of CRC GWAS loci.

#### 3.2.1. Ancestry

**Figure 1:**
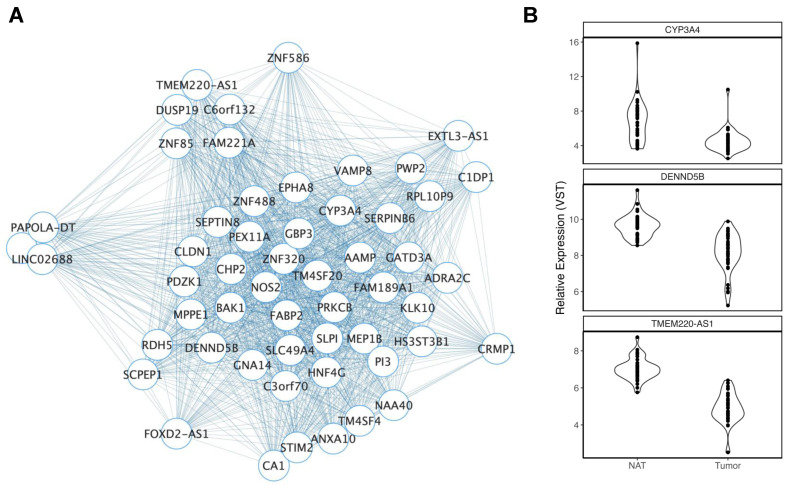
Overview of the palevioletred2 module. (**A**) Palevioletred2 module members are represented as nodes connected to other members through edges. The number of edges connected to each node provides a visual representation of the MM for each gene (node), where nodes with greater MMs display more connections. ME expressions of palevioletred2 were significantly greater in AA than EA colon stem cells. (**B**) Violin plots display significant differences in gene expression in TCGA-COAD of palevioletred2 module members that were mapped to CRC GWAS loci.

Four modules were nominally associated with self-reported measures of ancestry (*p* < 0.05). MM and GS were only significantly correlated in blue2 and palevioletred2 modules, with palevioletred2 also surviving FDR correction (FDR = 5.99 × 10^3^). This module consisted of 55 genes that displayed increased expressions in AA versus EA colon organoid stem-cell-enriched populations (Figure 1A). Of these, 25 were significant differentially expressed genes (DEGs) in our analysis of a TCGA-COAD tumor vs. NAT, including three that were mapped to known CRC GWAS genes (Figure 1B). However, most of these genes (*n* = 21) were reduced in tumors versus NAT. The blue2 module (Figure S5A) consisted of genes that were reduced in AA versus EA stem cells. Like palevioletred2, many blue2 genes were DEGs in our TCGA-COAD analysis (22 of 60 blue2 genes). However, the direction of effect was variable, with ~39% of genes found to be reduced in tumors (Figure S5B).

#### 3.2.2. Age

**Figure 2:**
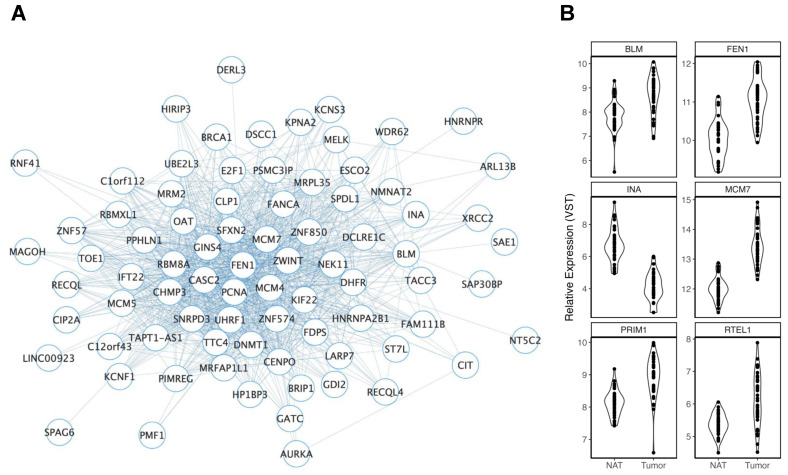
Overview of the lightpink3 module. (**A**) Lightpink3 module members are represented as nodes connected to other members through edges. Given the size of the module, a networking threshold of 0.1 was set to reduce poorly weighted connections prior to viewing in Cytoscape. The number of edges connected to each node provides a visual representation of the MM for each gene (node), where nodes with greater MMs display more connections. (**B**) Violin plots display significant differences in gene expression in TCGA-COAD of lightpink3 module members that were mapped to CRC GWAS loci.

Seven modules were associated with aging. Of those, skyblue4 (r = −0.74, FDR = 0.04), lightpink3 (r = −0.74, FDR = 0.04), and magenta4 (r = 0.73, FDR = 0.04) all survived FDR correction. Lightpink3 (Figure 2A) was a comparatively large module comprised of 396 genes, of which 54 were DEGs in TCGA-COAD and 6 (Figure 2B) were also genes mapped to CRC GWAS loci. The direction of effect of many of the CRC genes was variable in our analysis of tumors vs. NAT. However, this module was also significantly enriched for genes associated with telomere maintenance (GO:0000723, FDR = 1.70 × 10^3^), telomere maintenance via semi-conservative replication (GO:0032201, FDR = 4.90 × 10^3^), telomeric loop disassembly (GO:0090657, FDR = 0.013), and telomeric replication stress (the beginning and the end for the alternative lengthening of telomere cancers (FDR = 6.80 × 10^4^)), as well as Werner Syndrome (DOID:5688, FDR = 0.012). A nominal association between this module and smoking history (r = 0.52, *p* = 0.049) was also observed. Firebrick4 (r = −0.61, *p* = 0.02) consisted of 60 genes, of which 16 were reduced in CRC tumors. This module was enriched for genes previously associated with the effects of aging and cellular senescence in stem cells derived from bone marrow donors and hematopoietic progenitor cells [51].

Other FDR-corrected and nominal modules were associated with aging and consisted of TCGA-COAD DEGs that displayed a more consistent direction of effect. The skyblue4 module (r = −0.74, FDR = 0.04) contained 13 TCGA-COAD DEGs, of which 11 were also reduced in CRC tumors (Figure S6A,B). Magenta4 (r = 0.73, FDR = 0.04) contained 19 TCGA-COAD DEGs. Of these, 12 were increased in CRC tumors, including the CRC GWAS locus gene target, SNHG32 (Figure S7A,B). Of the nominally significant modules, lightsteelblue (r = 0.63, *p* = 0.01) and darkviolet (r = −0.57, *p* = 0.03) were of the most interest. The darkviolet module (r = −0.57, *p* = 0.03) was a relatively small module (*n* = 58 gene members). Despite this, 18 TCGA-COAD DEGs were present within this module, 15 of which were decreased in CRC tumors. This included ZNF132, a CRC GWAS locus gene target (Figure S8A,B). This module contained multiple genes that encode for zinc finger proteins and was primarily enriched for genes related to the regulation of transcription, DNA templating (GO:0006355, FDR = 1.53 × 10^12^), and the regulation of gene expression (GO:0010468, FDR = 5.28 × 10^12^).

**Figure 3:**
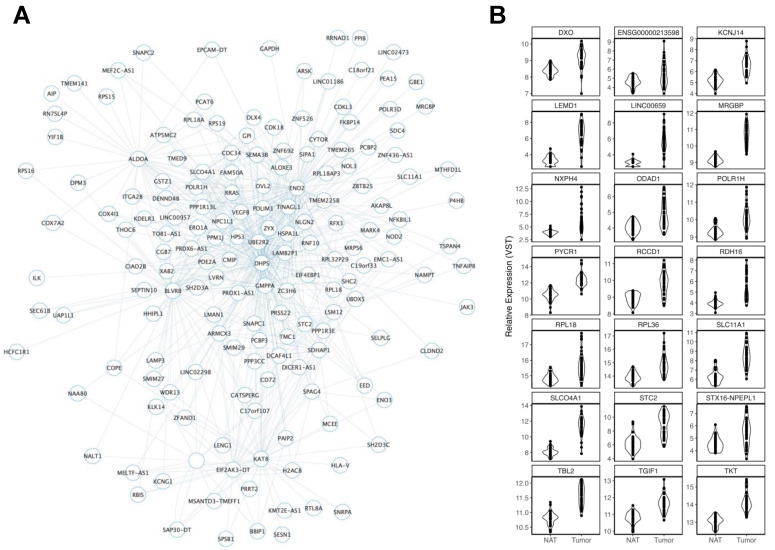
Overview of the lightsteelblue module. (**A**) Lightsteelblue module members are represented as nodes connected to other members through edges. ME expression was increased with increasing age. Given the size of the module, a networking threshold of 0.19 was set to reduce poorly weighted connections prior to viewing in Cytoscape. The number of edges connected to each node provides a visual representation of the MM for each gene (node), where nodes with greater MMs display more connections. (**B**) Violin plots display significant increases in gene expression in TCGA-COAD tumors of lightsteelblue module members that were mapped to CRC GWAS loci.

Lightsteelblue was a large module that consisted of 682 genes (Figure 3A), 111 of which were significant and increased in CRC tumors (111/185 TCGA-COAD DEGs, 60%). Twenty-one of these genes were mapped to CRC GWAS loci (Figure 3B). This module was also enriched for several relevant pathways, including the ATP metabolic process (GO:0046034, FDR = 4.27 × 10^5^), the generation of precursor metabolites and energy (GO:0006091, FDR = 6.67 × 10^5^), the glycolytic process (GO:0006096, FDR = 4.00 × 10^4^), and aerobic glycolysis (WP4629, FDR = 6.20 × 10^3^).

#### 3.2.3. Smoking History

**Figure 4:**
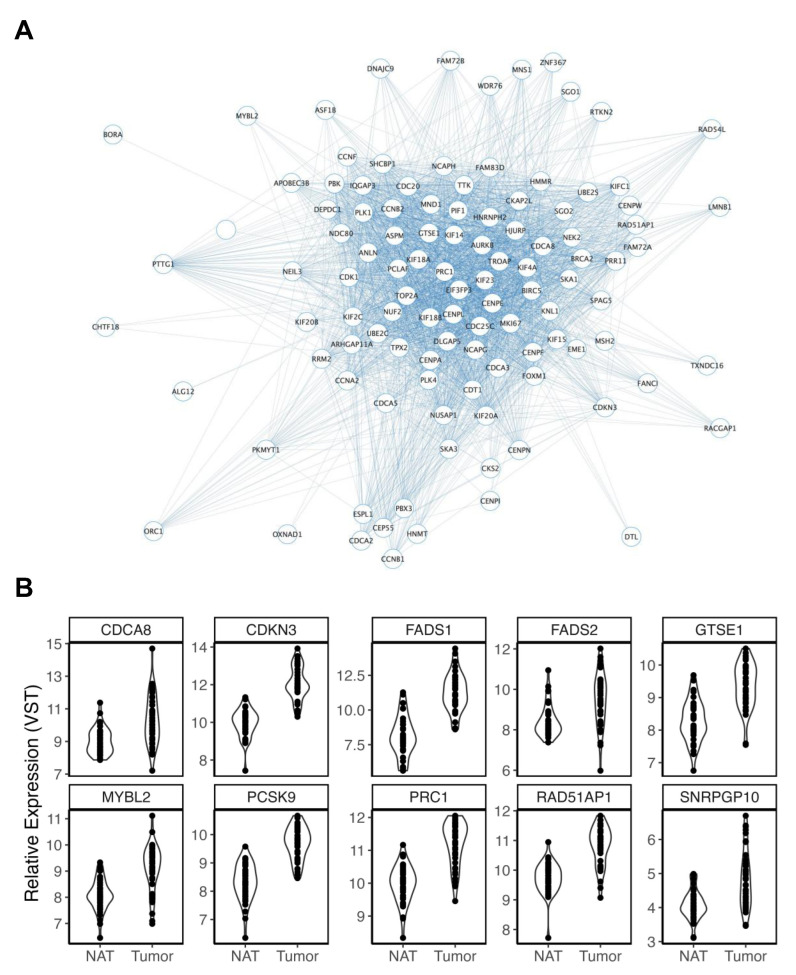
Overview of the purple module. (**A**) Purple module members are represented as nodes connected to other members through edges. ME expression was increased in former and current smokers. Given the size of the module, a networking threshold of 0.22 was set to reduce poorly weighted connections prior to viewing in Cytoscape. The number of edges connected to each node provides a visual representation of the MM for each gene (node), where nodes with greater MMs display more connections. (**B**) Violin plots display significant increases in gene expression in TCGA-COAD tumors of purple module members that were mapped to CRC GWAS loci.

Five modules were nominally associated with smoking history (*p* < 0.05). Lightpink3 was excluded, having been found to be more related to aging. Only salmon2 and purple modules were found to display significant correlations between their respective GS and MM scores and were, thus, considered for further interrogation. A pathway analysis of salmon2 revealed no relevant enrichments. The purple module consisted of 274 genes and represented the most significant module association with smoking (*p* = 6.00 × 10^3^) (Figure 4A). This module was enriched for genes previously associated with tobacco exposure in lung adenocarcinoma [52], as well as cell cycle (GO:0007049, FDR = 5.73 × 10^52^), signal transduction in response to DNA damage (GO:0042770, FDR = 2.93 × 10^7^), cellular response to DNA damage stimulus (GO:0006974, FDR = 2.00 × 10^6^), DNA damage response, and signal transduction by p53 class mediator (GO:0030330, FDR = 1.59 × 10^5^). Previously, our group published data on a 24 h exposure to smoking-related carcinogens in a large colon organoid dataset. Surprisingly, 174 of the 274 purple module members were also found to be nominally reduced in our initial carcinogen analysis [26]. One hundred and twenty-seven purple module members were also DEGs in TCGA-COAD, one hundred and nine of which were also increased in CRC tumors. Ten genes within this subset were also mapped to CRC GWAS loci (Figure 4B).

#### 3.2.4. Body Mass Index

**Figure 5:**
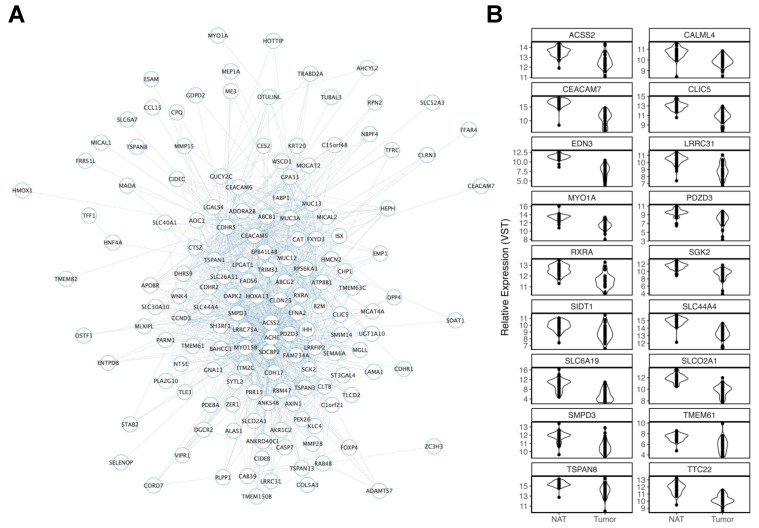
Overview of the darkred module. (**A**) Darkred module members are represented as nodes connected to other members through edges. ME expression was increased in former and current smokers. Given the size of the module, a networking threshold of 0.22 was set to reduce poorly weighted connections prior to viewing in Cytoscape. The number of edges connected to each node provides a visual representation of the MM for each gene (node), where nodes with greater MMs display more connections. (**B**) Violin plots display significant decreases in gene expression in TCGA-COAD tumors of darkred module members that were mapped to CRC GWAS loci.

We identified two modules nominally associated with increasing BMI. MEs for both darkolivegreen2 (r = −0.58; *p* = 0.02) (Figure S9) and darkred (r = −0.53; *p* = 0.02) (Figure 5A) were negatively correlated with increasing BMI. A pathway enrichment analysis revealed highly relevant enrichments for the lipid metabolic process (GO:0006629, FDR = 9.35 × 10^6^), the cholesterol biosynthetic process (GO:0006695, FDR = 0.018), and fat digestion and absorption (hsa04975, FDR = 0.048) in darkred module members. A total of 210 genes within the darkred module passed FDR correction in TCGA-COAD, with 187 (89.05%) being reduced in tumors versus NAT. This included 18 (of 22 total) gene targets of CRC GWAS loci (Figure 5B). Of these, TSPAN8, PDZD3, SMPD3, CLIC5, and RXRA displayed high MMs (r = 94, r = 0.91, r = 0.89, r = 0.88, and r = 0.87, respectively).

#### 3.2.5. Biological Sex

Three modules were nominally associated with self-reported biological sex: lightcoral, plum3, and lightcyan1 (Figures S10–S12). The latter two consisted of genes displaying increased expressions in males. Lightcyan1 was significantly enriched for RNA binding (GO:0003723, FDR = 0.043). However, no other enrichments were found across any of these three modules.

## 4. Discussion

In this study, we present a proof-of-principle pilot study that demonstrates the utility of WGCNA in the assessment of CRC risk factors in stem cells derived from healthy colon organoids. Identifying mechanisms attributed to risk may lead to the identification of novel targets for drug discovery and identify biomarkers to support precise preventive measures in asymptomatic populations [53]. Such early markers may also inform patients in a way to make effective lifestyle changes for modifiable factors [53,54]. Stem cell profiling represents a strong platform for the discovery of targetable molecular differences occurring in at-risk populations [55] by assessing differences occurring in long-lasting cell populations that are believed to be highly relevant to CRC initiation [16]. In CRC, stem cells preferentially activate various signaling pathways that are important for cellular maintenance and self-renewal. These pathways include, but are not limited to, Wnt/β-Catenin, BMP, Notch, TGF-β, and Hedgehog [56]. Mutations affecting genes within these pathways have been shown to increase CRC risk [57,58,59]. Lgr5^+^ stem cells are also hypothesized to be the origin cells for CRC [16,55]. Thus, further interrogation of the role of risk factors on colon stem cells is warranted. Our group has previously used WGCNA to identify robust mechanisms through which CRC risk factors may exert their effects [24,26,28]. WGCNA has also been increasingly used as a method to identify drivers of gene correlation structures and their relationships to CRC outcomes [60,61], as well as differences across subgroups [28]. By overlaying our WGCNA findings with expression data comparing CRC patient tumors to NAT [43], we were able to contextualize mechanisms through which lifestyle and demographic factors impacted coordinated gene expression modules in the framework of CRC. Through additional overlay with known CRC GWAS loci, we provided novel and strong evidence for roles in CRC risk for a number of genes that appeared highly relevant to risk module structure, such as *TSPAN8*, *PDZD3*, *SMPD3*, *CLIC5*, and *RXRA*. This subset of genes presented plausible biological mechanisms through which environmental factors exerted their effects and should be prioritized for future functional follow-up studies.

CRC is an age-related disease, with approximately 90% of cases attributed to individuals 50 years or older [62]. While aging is the strongest risk factor for CRC development, the biological mechanisms underpinning this relationship have yet to be fully explained [63]. However, several mechanisms have been proposed, including (1) epigenetic drift [64], (2) cellular senescence [65], and (3) metabolic reprogramming [66]. Epigenetic drift is a process of reduced stability occurring in epigenetic marks over successive cell divisions, and DNA methylation analyses of cohort studies have revealed strong overlaps between sites associated with cancer and those observed in aging [64,67]. However, neither study has considered the effects of these differences in DNA methylation on nearby gene expression. Cellular senescence describes a process through which stem cells reach a pre-defined limit of cellular divisions [68], leading to stable cell cycle arrest, mitochondrial dysfunction, and metabolic abnormality [69]. This process occurs naturally through aging but can also be accelerated by other factors, including reactive oxygen species, telomere shortening, and alterations to tumor suppressor gene function [69].

Our analysis of aging revealed the presence of two types of modules. The first type consisted of modules related strongly to aging with little obvious relationship to CRC. Lightpink3 represents a good example of this type of module. The biological plausibility of the lightpink3 module was apparent. It was heavily enriched for telomeric processes, which are widely reported to be altered as a consequence of aging [70]. Similarly, firebrick4 was enriched for genes previously associated with cellular senescence in other stem cell populations [51] These data provide evidence to support the power of a WGCNA for capturing biological variation within a colon organoid model. The second type of module consisted of genes that displayed strong similarities to differences observed between CRC tumors and NAT. Both lightsteelblue and darkviolet provided strong evidence to support mechanisms through which aging drove CRC risk. The darkviolet module consisted of genes that were negatively correlated with aging. The vast majority (83.33%) of TCGA-COAD DEGs that were also darkviolet module members were reduced in CRC tumors. This included *ZNF132*, a gene previously associated with CRC through GWAS. This gene was previously found to decrease with age in other cohorts and tissues [71] and was found to act as a tumor suppressor gene in esophageal squamous cell carcinoma [72]. Reduced expression of *ZNF132* may, therefore, represent one important mechanism through aging increasing CRC risk.

Lightsteelblue consisted of genes that were predominately positively correlated with aging. Over 100 lightsteelblue module members also displayed increased expressions in CRC tumors. This module was heavily enriched for altered metabolic processes, including glycolysis. Interestingly, previous research on *C. elegans*, a genetic model of aging, found that aged organisms shifted from aerobic to less efficient anaerobic glycolysis [73]. Similarly, research in *Drosophila* showed that the intestinal stem cells of older flies underwent metabolic reprogramming toward aerobic glycolysis to drive stem cell proliferation and eventual hyperplasia [66]. The authors highlight that this transition was similar to the oncogenic transformation seen in intestinal stem cells that express Ras^V12^, proposing a “Warburg-like” effect for older cells. Older stem cells failed to uptake calcium ions from the mitochondria, a process deemed critical for the transition from quiescence to proliferation in younger cells. As such, they remained chronically active. Though enrichments for RAS signaling were not observed within this module, lightsteelblue did contain some *RAS*-related genes, including *RRAS* and *RASA4CP*. Further, we identified 21 genes associated with CRC through both GWAS and TCGA-COAD. Increased expressions of *SLC11A1* [74], *STC2* [75], *NXPH4* [76], and *TKT* [77] have all been associated with increasing glycolysis rates that contribute to the establishment of various disease phenotypes. Additionally, the silencing of another CRC GWAS gene, *PYCR1*, also led to a switch from anaerobic glycolysis to oxidative phosphorylation in mouse mesenchymal stem cells [78]. In summary, the lightsteelblue module brought together findings derived from in vivo experiments of stem cells that revealed the important metabolic effects of aging with findings from in vitro experiments across cancer cell lines, as well as with data from CRC tumors and GWAS, to not only strengthen the hypothesis that a shift to aerobic glycolysis drives CRC risk in aged cells, but also to prioritize targets that may be driving this shift. Whether these differences occur earlier in EOCRC represents an important question for future consideration.

Cigarette smoking is consistently linked to increased risk for multiple cancers, including CRC [79]. Our purple module consisted of genes that were increased in colon stem cells of smokers vs. previous smokers. This module was enriched for genes previously associated with tobacco smoke, confirming that it captured biological signals attributed to smoking. It was also enriched for multiple pathways related to the cell cycle and DNA damage and was comprised of over 100 genes that were overexpressed in CRC tumors. Indeed, of the eight CRC GWAS loci found within this subset of CRC-related smoking genes, *CDCA8* [80], *CDKN3* [81], *FADS1* [82], *FADS2* [83], *MYBL2* [84], *PCSK9* [85], and *PRC1* [86], have all been reported to be overexpressed in others cancers and to play important roles in the DNA damage response pathway. Previous research showed that *MYBL2* was upregulated by lung adenocarcinoma tumors to serve as an important driver of error-prone DNA repair [84], indicating an important role for this gene in facilitating the progression of other cancers. In line with this finding, CRC tumor overexpression of *MYBL2* was found to be within the top 10% of all DEGs identified in our analysis of TCGA-COAD, the second most significant within the purple module (only *PCSK9* was more significantly associated with CRC), and within the top 35% of most connected genes within the module. *MYBL2* has also been shown to have direct effects on other key purple module members, such as *CDCA8*. In ovarian cancer cells, *MYBL2* was shown to serve as an upstream transcription factor to *CDCA8* [80], directly increasing its expression to alter cell cycle regulation and DNA repair. Further, while *PRC1* displayed a greater module membership with the purple module, this gene was also found to be a downstream target of *MYBL2* in other cancer cell lines [87]. Larger studies in more well-characterized samples may be able to determine the relationship between these differences and variables, such as smoking years, as well as whether the effects are reversible following smoking cessation. Despite this, these findings allude to a highly coordinated mechanism through which smoking increases the risk of CRC by increasing *MYBL2* expression to drive additional plausible CRC targets, culminating in altered DNA damage response and cell cycle pathways.

U.S. population levels of BMI have increased consistently over recent decades, and a growing body of evidence implicates this shift as an important risk factor contributing to the rising rates of EOCRC [88]. Here, our analysis identified two modules of gene co-expression that were significantly associated with BMI. Of these, the darkred module was of most interest, given that genes contained within were enriched for multiple metabolic, lipid, and cholesterol pathways. By overlaying genes found within this module with data from TCGA-COAD and CRC GWAS, we found that *RXRA* was one of 18 module members that was a target of CRC GWAS loci and was significantly reduced in colon tumors. *RXRA* was also highly relevant to module structure and displayed the highest independent association with BMI in our analysis. This gene encodes for Retinoid X receptor alpha, a member of a nuclear receptor superfamily that functions as transcription factors modulating various processes, including metabolism [89]. Studies of obese, insulin-resistant rats have also shown that activating the retinoid receptor reduces appetite and that an intracerebroventricular injection of a retinoid receptor agonist helps to regulate energy balance, leading to reductions in insulin and BMI gain [89,90]. *RXRA*, therefore, represents a biologically plausible target through which increased BMI may affect metabolic pathways in colon stem cells to increase CRC risk.

A major strength of this study was the inclusion of organoids from individuals of two self-reported ancestry groups. Racial disparities exist for CRC, and AA individuals tend to present with more advanced disease at an earlier age and have poorer clinical outcomes [30]. This is further compounded by a gross data collection disparity, with limited omics-level data derived from AA populations [91]. Such disparities increase the risk of falsely attributing EA-specific disease mechanisms to AA populations. In recent years, calls for greater minority representation in omics studies have been made [92], and it has become increasingly clear that, to better define mechanisms attributed to CRC risk, greater racial diversity of study cohorts is imperative [93]. Our WGCNA identified two ancestry-specific modules containing genes significantly associated with CRC through an expression analysis of TCGA-COAD. We reasoned that, as AAs have an increased lifetime risk for right-sided colon cancer, modules characterized by increased expressions of genes in AA versus EA stem cells would be comprised of genes overexpressed in CRC tumors. Surprisingly, the direction of effect within these modules was discordant to that expected prior to our analysis. For example, palevioletred2 module members were increased in AA vs. EA individuals. However, many genes within this module were found to be reduced in CRC tumors. The reasons for this discrepancy were unclear. However, this ancestry study was based on relatively small sample sizes, and larger sample sizes, data on the socioeconomic status of individuals in future studies, and the coupling of findings with patient outcomes at follow-up may help to improve insight. Despite this, the biracial nature of our datasets provided important representation to the analysis of the other CRC risk factors considered within this study. It should be noted that we were able to combine both AA and EA populations for our assessment of other CRC risk factors, greatly strengthening our study.

There are several factors that should be considered in the interpretation of our findings. Nearly 10% of U.S. Blacks are immigrants [94]. Here, the sample size, lack of genotyping data, and lack of information for ancestral heritage required that all Black individuals were analyzed as one group: AAs. This could lead to false positives and/or negatives driven by a failure to account for distinct biological, cultural, and/or socioeconomic differences that may be present [95]. We specifically focused on organoids generated from the right colon. This limited the scope of our findings, especially given that our group has previously revealed side-specific differences between normal colon organoid responses to various CRC risk factors [22,24,25]. However, this region was chosen given the increased disparities between AA and EA individuals in right-colon cancer. Further, we identified genes that were differentially expressed in both CRC tumors and in association with CRC risk factors in healthy colon stem cells. However, our patient population consisted of sampling at one timepoint. Additional patient follow-ups coupled with collections of samples at multiple timepoints would allow for increased power to detect differences in patient outcomes. Similarly, CRC is a heterogenous disease. Thus, larger and more racially diverse CRC datasets are important for future validations. Furthermore, some modules were not related to any of the five traits considered in our analysis. However, it is possible that coupling this approach with more extensive epidemiological and demographic information may reveal unique insight into other factors affecting risk. Additionally, many of the modules identified here represent biologically plausible mechanisms that may drive lifestyle/environmental components of CRC risk. However, our dataset was small, and many associations did not pass FDR correction. Larger study sizes can also help to better define these associations and the robustness of the co-expression observed within each module. Increasing sample size may be particularly important when defining the effects of continuous traits or associations between unbalanced subgroups. Improving sample size would additionally allow for less strict filtering of gene expression prior to WGCNA, as spurious variation in lowly expressed genes would be less impactful on the correlation structure of larger datasets. The strict filtering here was performed given the limited sample sizes. However, it may have led to the removal of true biological signals, for example from genes that were highly expressed in one binary phenotype, such as those that were X-linked. Importantly, the power of WGCNA to detect biologically relevant differences in gene co-expression was influenced by sample size and the overall cleanliness of the data. Studies may consider the use of methods such as SVA to mitigate any perceived batch effects prior to network generation [96]. However, our principal component analysis did not reveal any obvious batch effects. Finally, the CV medium was enriched for LGR5+ stem cells; however, they were not pure. Coupling the use of CV medium with fluorescence-activated cell sorting (FACS) prior to sequencing may produce an even cleaner signal, although this would require substantially more wells per sample to be grown and may not be appropriate for large-scale approaches. Additionally, scRNA-seq would allow for the simultaneous analysis of multiple cell populations. However, the current cost of single-cell sequencing mostly prohibits the study of larger-scale designs for many studies.

Despite these limitations, this proof-of-principle study revealed novel insights into the complex relationships between healthy colon stem cells and lifestyle factors. Importantly, the biracial nature of this dataset helped to address key racial and data availability disparities for AA populations. Indeed, our work is in line with calls for action from a recently published review that highlighted the need for additional consideration of underserved communities in preclinical research [97]. This would lead to more targeted approaches in the future. In the future, combining WGCNA from purified stem cell populations of larger, well-characterized, and diverse cohorts may provide new insights into the molecular effects of an individual’s exposome and its relationship to risk. Finally, CRC is a highly heterogenous disease consisting of multiple subtypes. Our group previously highlighted the power of WGCNA in CRC tumors to improve understanding of the gene–gene relationships of known and novel drivers of these subgroups [28]. However, such work remains limited by cellular heterogeneity, which requires extensive correction [28], or risks convoluting gene co-expression networks with cell-specific signals observed across multiple cell populations. Combining WGCNA with organoids derived from CRC tumors may provide additional insight into cell-specific relationships to clinical outcomes.

## 5. Conclusions

In conclusion, by enriching for LGR5+ stem cell populations, we identified robust modules related to CRC risk factors in a cell population highly relevant to CRC initiation. Our use of a racially diverse patient cohort increased the validity and relevance of our findings to the general population. Future studies should continue to expand upon this work to gain further insight into CRC racial disparities. Through analysis of CRC tumor and GWAS data, we prioritized drivers of mechanisms through which effects on risk may be exerted. *RXRA* and *MYBL2* represented exciting targets through which BMI and cigarette smoking, respectively, altered expression to modulate risk. Further, the metabolic reprogramming of stem cells throughout aging represented one factor through which aging may increase CRC risk. Our analysis of the lightsteelblue module revealed several highly connected module members related to metabolic reprogramming that were identified in CRC both through an analysis of tumors and CRC GWAS loci. Together, our findings highlight numerous targets for further functional follow-up of CRC risk.

## Figures and Tables

**Table 1:** Summary of individual-level data collected for each sample. Age and BMI represent values at the time of colonoscopy. Self-reported smoking history was collected for individuals and binarized as those who had never (0) and ever (1) smoked.

Deidentified ID	Sex	Ancestry	Age	Smoking History	BMI
P1055R	F	EA	22	0	26.4
P1081R	M	EA	66	0	22.96
P1096R	M	EA	53	0	50.4
P1099R	F	AA	56	0	42.4
P1112R	F	EA	50	0	21.6
P1121R	F	AA	52	0	27.72
P1148R	F	EA	59	1	25.52
P1176R	M	AA	69	0	23.9
P1229R	F	EA	53	0	27.79
P1239R	M	AA	50	0	32.32
P1246R	F	AA	54	1	47.77
P1252R	F	AA	31	1	33.78
P1263R	F	EA	62	0	49.86
P1268R	F	AA	58	0	39.6
P1270R	F	AA	60	0	28.1

**Table 2:** Summary of significant associations between ME values of each module and CRC risk factors. GS vs. MM represents the summary of the correlation analysis between the two and serves as a strong indicator of quality control. Negative r values are indicative of a negative relationship between the ME of a module and a trait of interest.

Trait	Module	GS vs. MM r	GS vs. MM P	r	*p*	FDR
Age	skyblue4	0.49	5.30 × 10^4^	−0.74	1.46 × 10^3^	0.04
Age	lightpink3	0.69	<2.20 × 10^16^	−0.74	1.50 × 10^3^	0.04
Age	magenta4	0.49	1.90 × 10^4^	0.73	1.94 × 10^3^	0.04
Age	coral3	0.4	1.40E × 10^6^	0.64	0.01	0.14
Age	lightsteelblue	0.49	<2.20 × 10^16^	0.63	0.01	0.14
Age	firebrick4	0.42	8.60 × 10^4^	−0.61	0.02	0.15
Age	darkviolet	0.42	1.20 × 10^3^	−0.57	0.03	0.21
BMI	darkolivegreen2	0.34	1.10 × 10^4^	−0.58	0.02	0.63
BMI	darkred	0.39	1.90 × 10^15^	−0.53	0.04	0.63
Ancestry	palevioletred2	0.55	1.20 × 10^5^	0.84	1.07 × 10^4^	5.99 × 10^3^
Ancestry	blue2	0.51	2.60 × 10^5^	−0.66	6.91 × 10^3^	0.18
Smoking History	purple	0.6	<2.20 × 10^16^	0.67	6.00 × 10^3^	0.34
Smoking History	salmon2	0.29	0.03	−0.53	0.04	0.56
Sex	lightcoral	0.45	2.50 × 10^4^	−0.71	3.01 × 10^3^	0.17
Sex	plum3	0.29	0.03	0.57	0.03	0.54
Sex	lightcyan1	0.35	1.80 × 10^7^	0.56	0.03	0.54

## Data Availability

Raw fastq files and pre-processed count matrix data have been uploaded to Gene Expression Omnibus under accession number GSE230067.

## Supplementary Materials

The following are available online at https://www.mdpi.com/article/10.3390/cancers15143550/s1, Figure S1: Principal component analysis of sample cohort, Figure S2: Summary of regression analysis on cell score between matched samples grown in complex (standard organoid media) and CV (stem cell enriching media) conditions, Figure S3: Summary of qPCR analysis used to validate stem cell enrichment, Figure S4: Overview of WGCNA, Figure S5: Overview of the blue2 module, Figure S6: Overview of the skyblue4 module, Figure S7: Overview of the magenta4 module, Figure S8: Overview of the darkviolet module, Figure S9: Overview of the darkolivegreen2 module, Figure S10: Overview of the lightcoral module. Figure S11: Overview of the plum3 module, Figure S12: Overview of the lightcyan1 module, Table S1: Summary mapping statistics for RNA-seq data, Table S2: Summary statistics following regression of TCGA-COAD combined with WGCNA gene module information of normal colon organoids.
