# Supplementary material for: Assessment of Colorectal Cancer Risk Factors through the Application of Network-Based Approaches in a Racially Diverse Cohort of Colon Organoid Stem Cells
Source: Cancers (Basel). 2023 Jul 9;15(14):3550. doi: 10.3390/cancers15143550 (PMC10377524; doi:10.3390/cancers15143550)
Supplement: Supplementary file 1 [file cancers-15-03550-s001.zip › Supplemental.pdf]

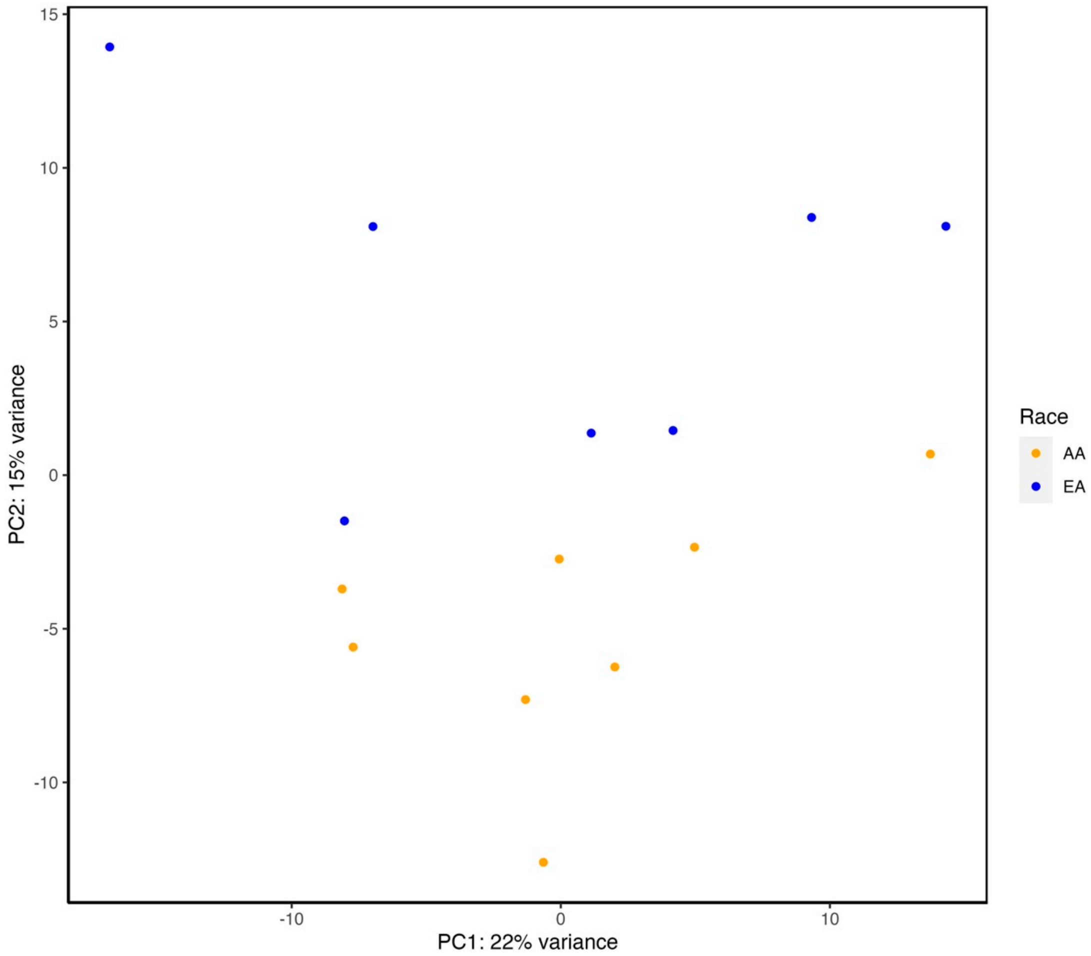

Figure S1: Principal component analysis of sample cohort. Variance-stabilizing transformation was carried out on gene expression data and principal components were generated using the plotPCA function of DESeq2. Principal component values were manually imported into ggplot2 and samples were color coordinated, with orange and blue corresponding to individuals who self-report as AA and EA, respectively. Visual inspection of plots revealed that self-reported ancestry was adequately able to stratify samples along the second principal component.

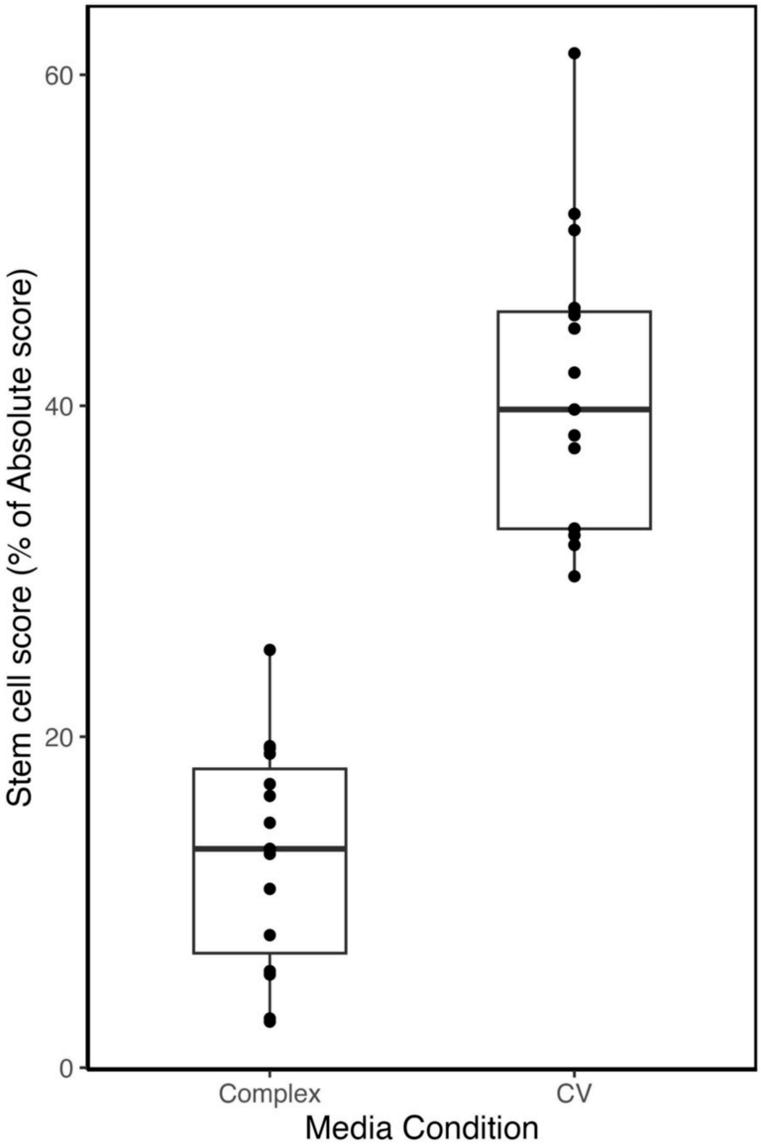

Figure S2: Summary of regression analysis on cell score between matched samples grown in complex (standard organoid media) and CV (stem cell enriching media) conditions. Absolute scores were generated in CIBERSORTx for regression modelling. Scores were then displayed as a percentage of total cellular content for visualization.

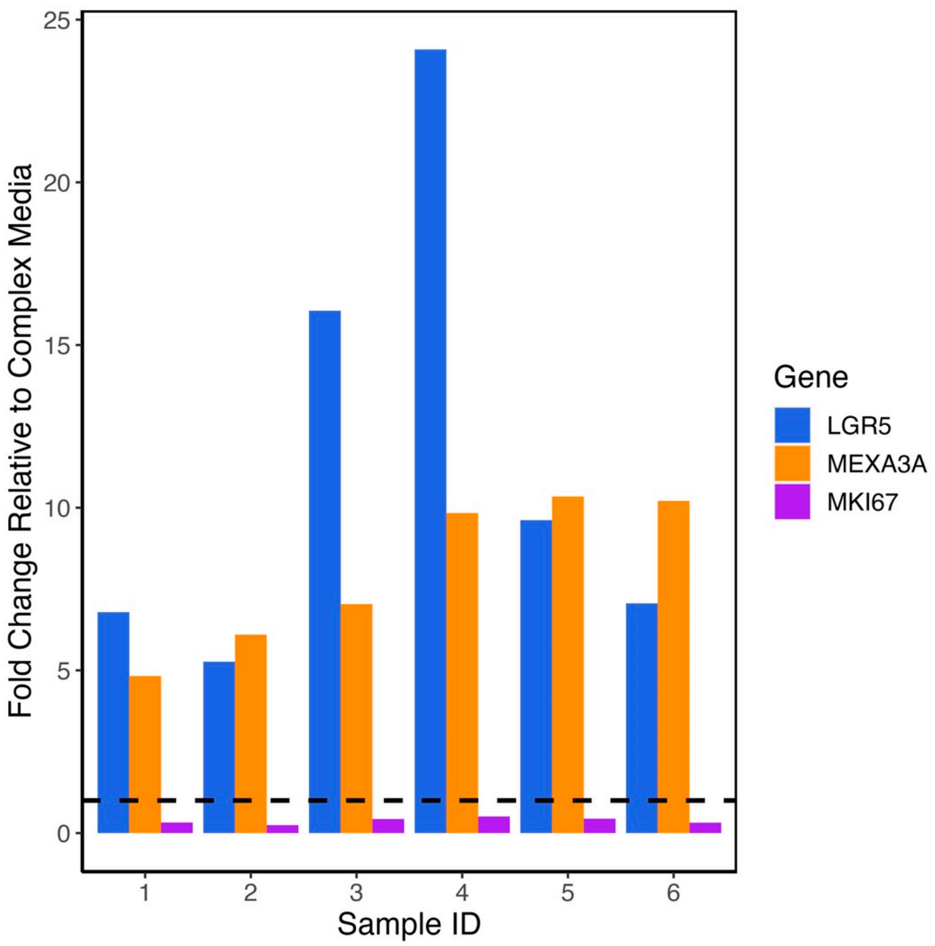

Figure S3: Summary of qPCR analysis used to validate stem cell enrichment. RNA remaining following RNA-sequencing of six colon organoid lines were grown in CV and complex media was reverse-transcribed and qPCR was performed on two markers of stem cells (*LGR5* and *MEX3A*) as well as one marker of overall proliferation (*MKI67*). Fold change estimates were calculated through the delta-delta CT method by first normalizing to a control gene (*GUSB*) before comparing CT values to complex media. CT values were then transformed to obtain fold change estimates relative to complex media. Values less than FC=1 (black dashed line) are indicative of a reduction in expression in CV media. As seen in our RNA-sequencing analysis, stem cell markers were highly expressed in CV versus complex media. These markers also appeared to show a reduced proliferative capacity, as shown by the reduction in *MKI67*.

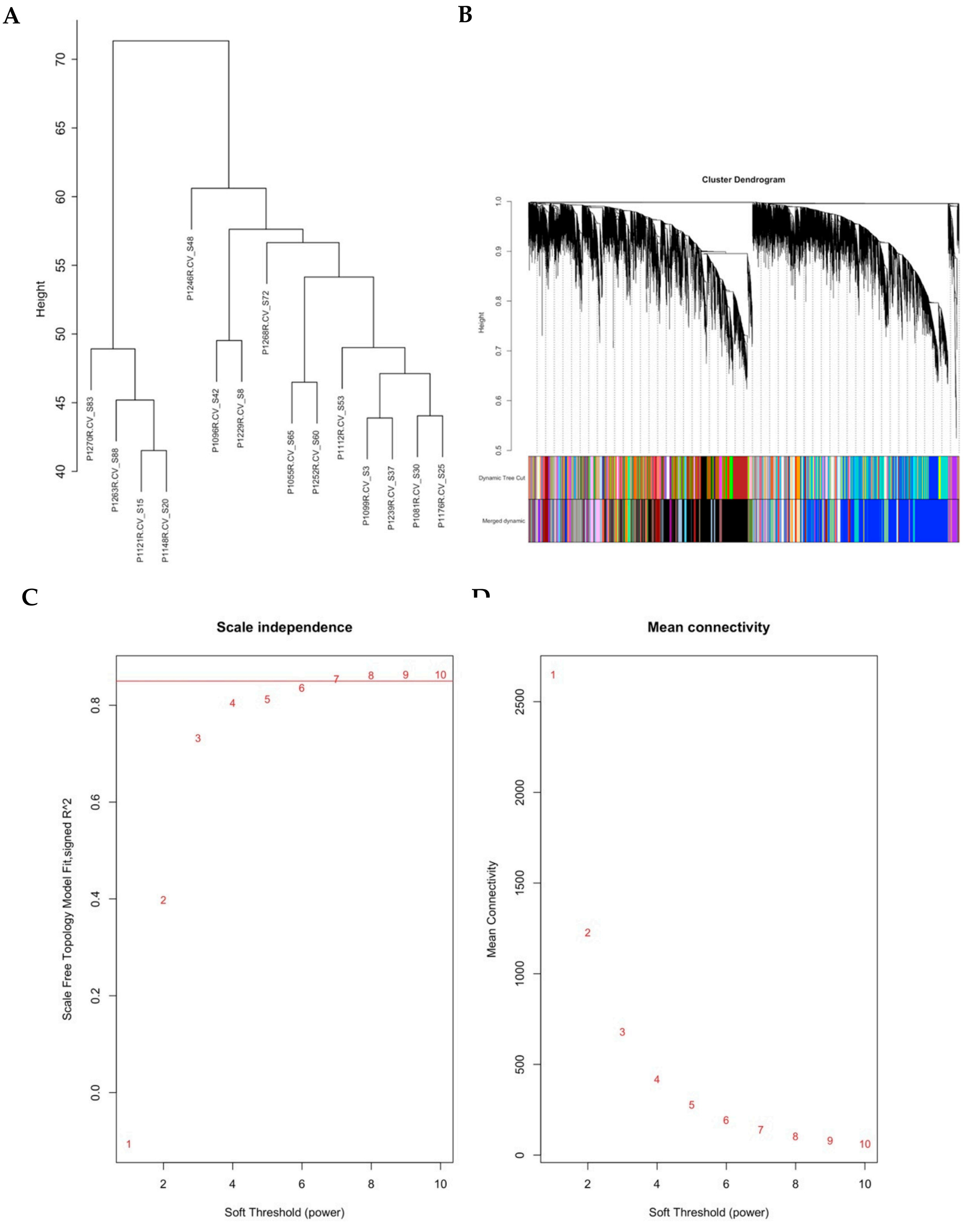

Figure S4: Overview of WGCNA. (A) Hierarchical clustering was used to determine the relationship between samples based upon overall gene expression. No obvious outliers were detected. B) Highly related modules were merged using a correlation cut-off of r=0.8. "Merged dynamic" modules were considered for downstream analysis. C) Analysis of scale-free topology under increasing soft-thresholding powers. A soft-thresholding power of 7 was the first power begin to "elbow" at an approximate scale-free topology (R^2=0.85). D) Plot showing the mean connectivity change of the network as a function of altering soft-thresholds.

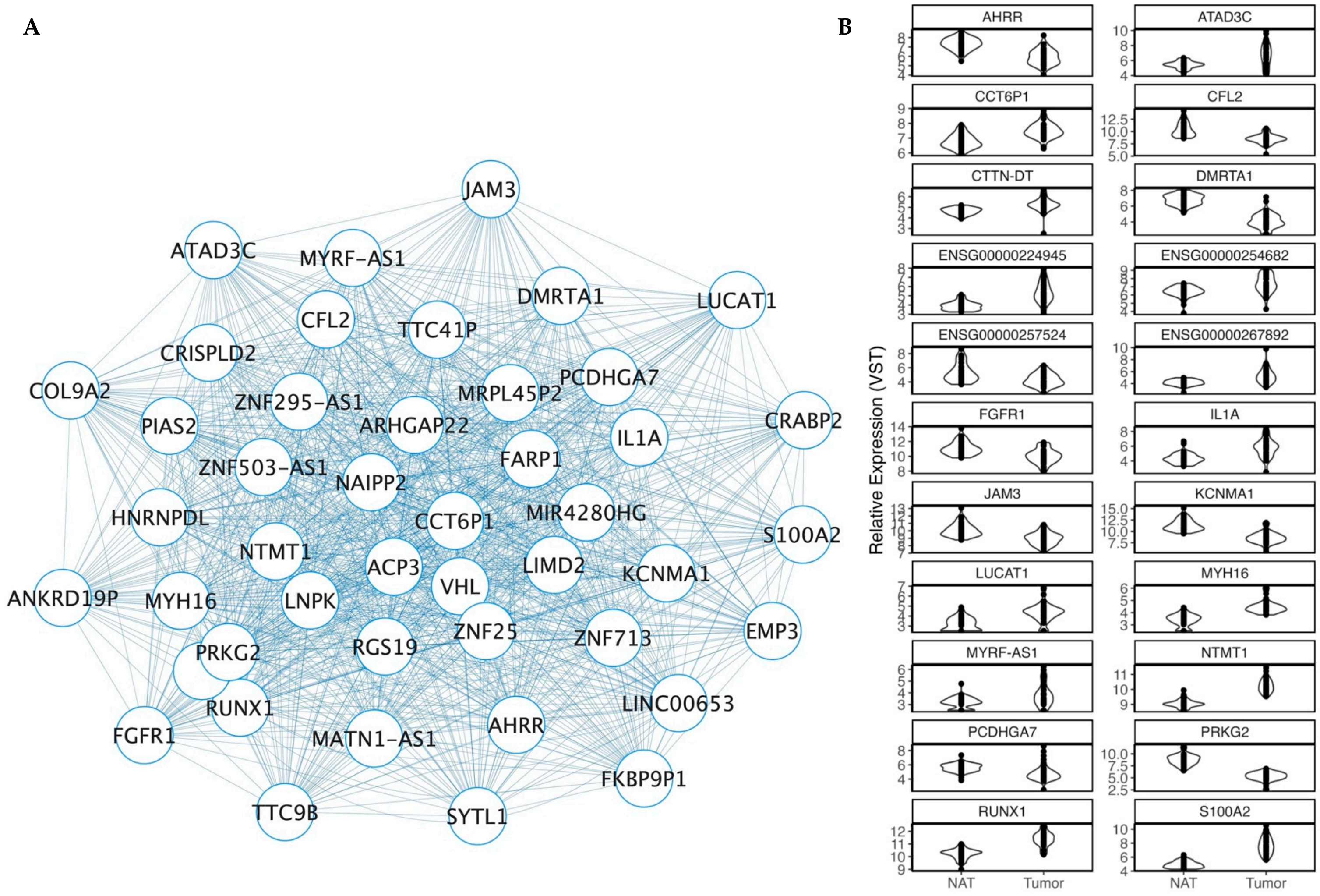

Figure S5: Overview of the blue2 module. A) Blue2 module members are represented as nodes connected to other members through edges. ME expression was increased in EA. B) Violin plots displaying the significant differences in gene expression in TCGA-COAD of blue2 module members.

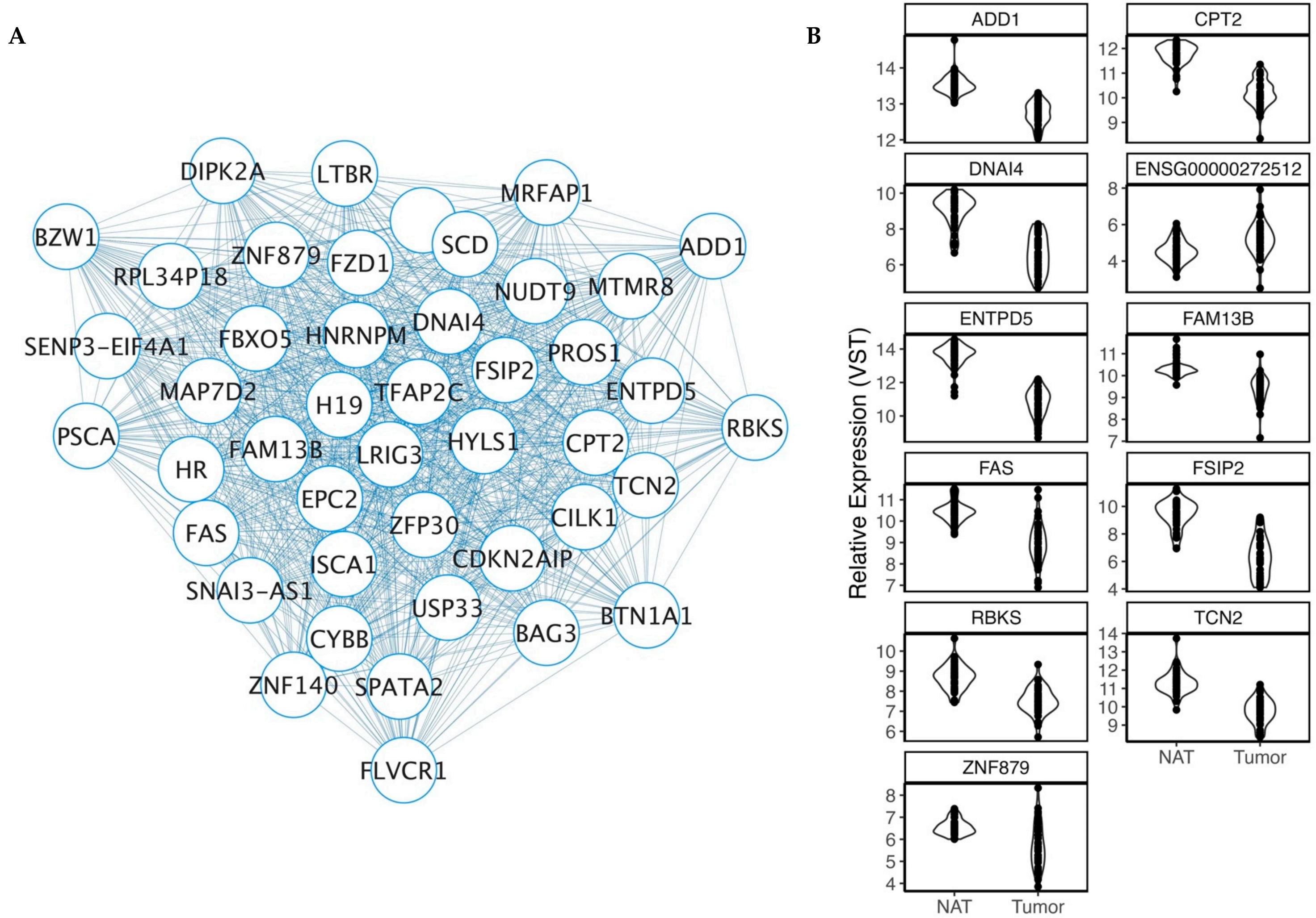

Figure S6: Overview of the skyblue4 module. ME expression decreased with increasing age. A) Skyblue4 module members are represented as nodes connected to other members through edges. B) Summary of DEG analysis of skyblue4 module members found to be significantly reduced in TCGA-COAD tumors.

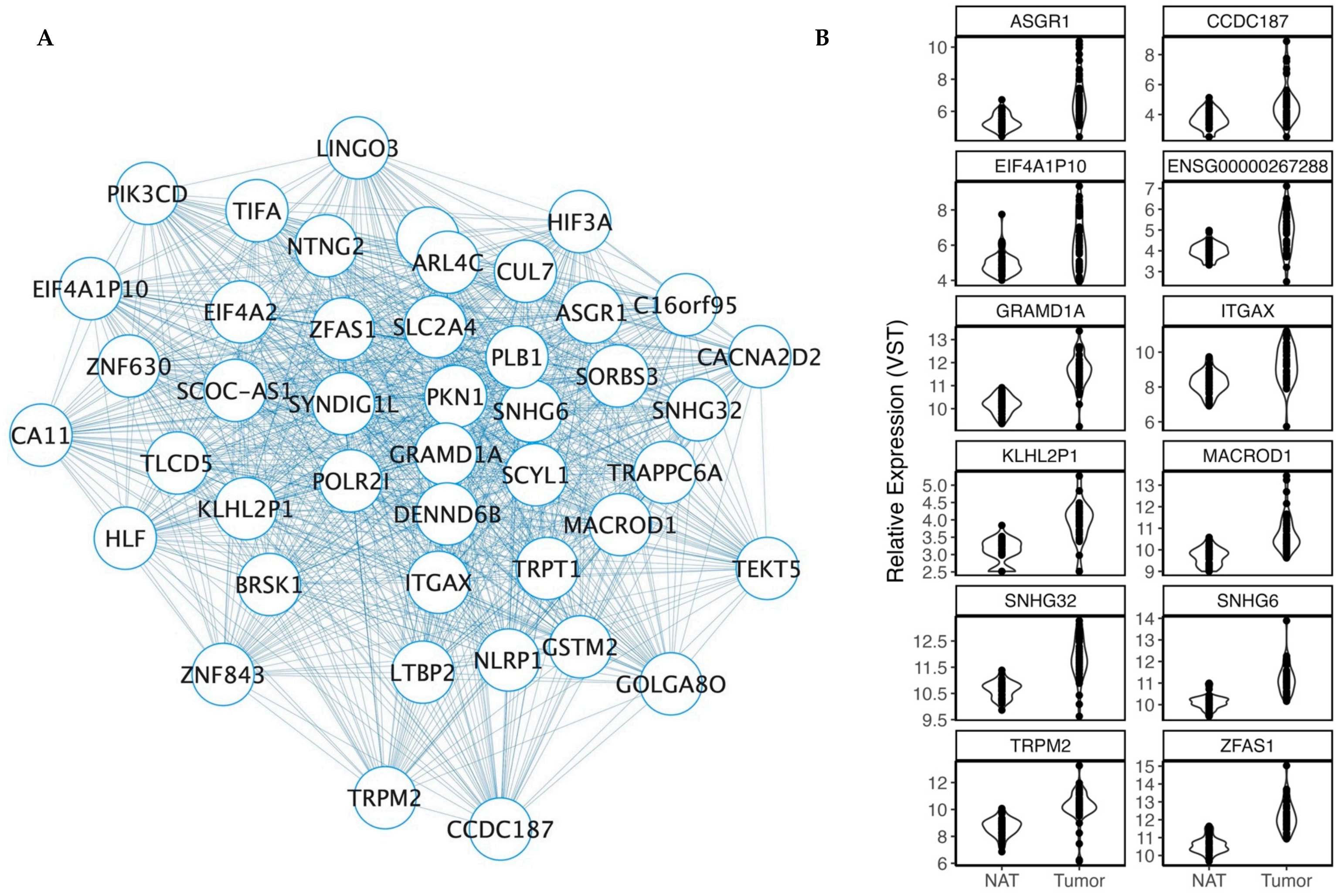

Figure S7: Overview of the magenta4 module. ME expression increased with increasing age. A) Magenta4 module members are represented as nodes connected to other members through edges. B) Summary of DEG analysis in TCGA-COAD of magenta4 module members that are increased in CRC tumors.

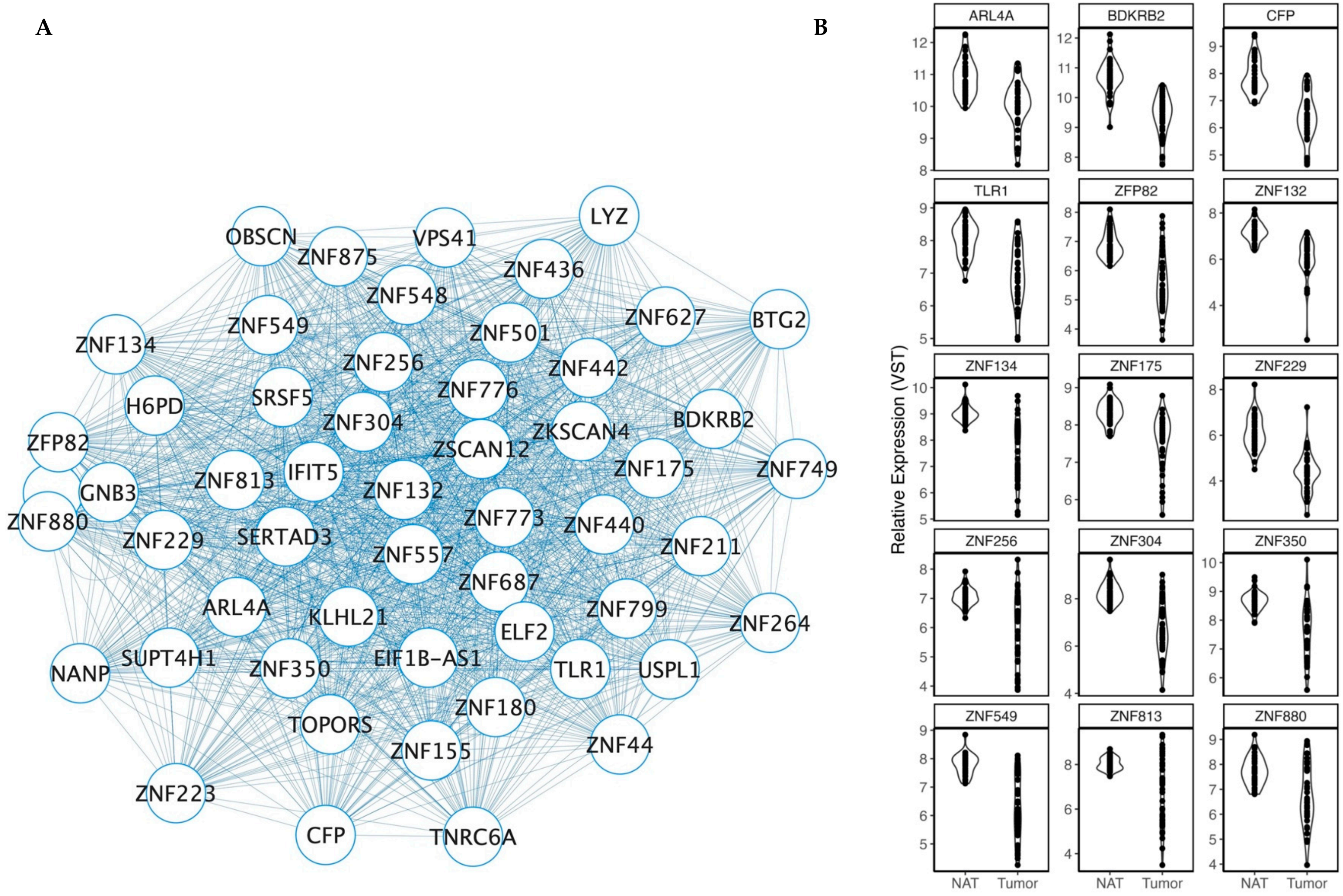

Figure S8: Overview of the darkviolet module. ME expression decreased with increasing age. A) Darkviolet module members are represented as nodes connected to other members through edges. B) Summary of DEG analysis in TCGA-COAD of darkviolet module members that are decreased in CRC tumors.

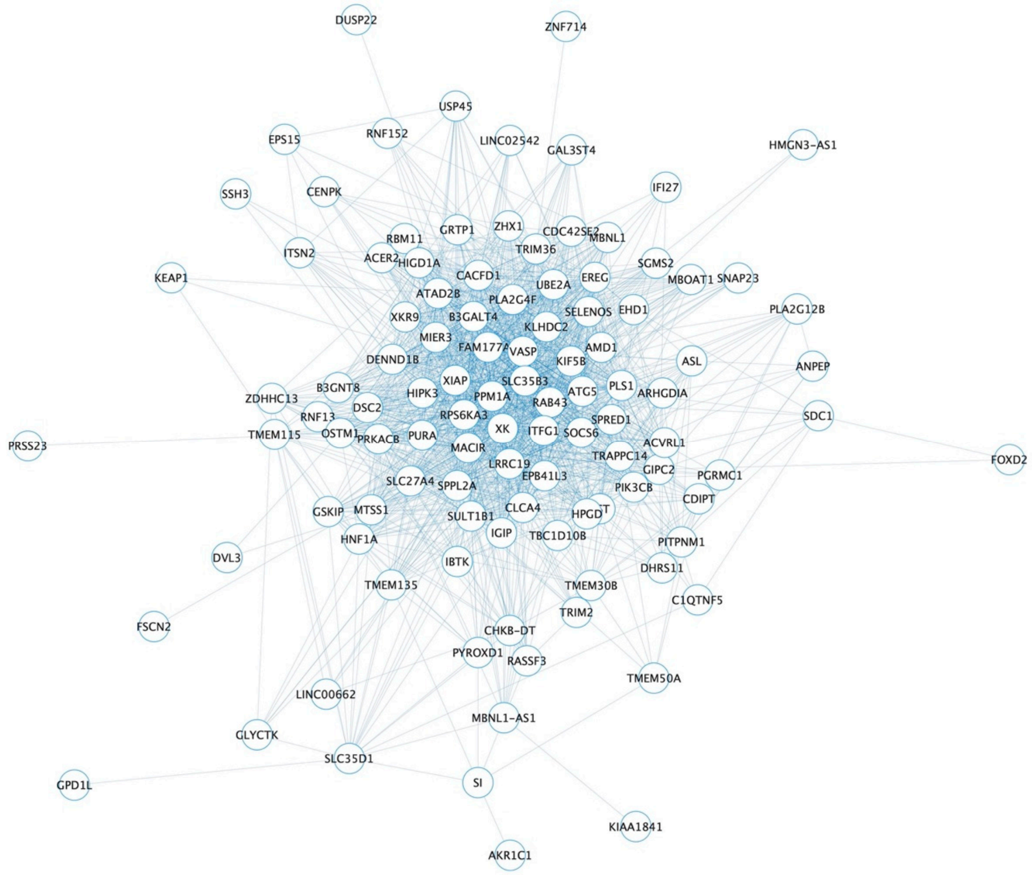

Figure S9: Overview of the darkolivegreen2 module. ME expression decreased with increasing BMI. Given the size of the module, a networking threshold of 0.05 was set to reduce poorly weighted connections prior to viewing in Cytoscape.

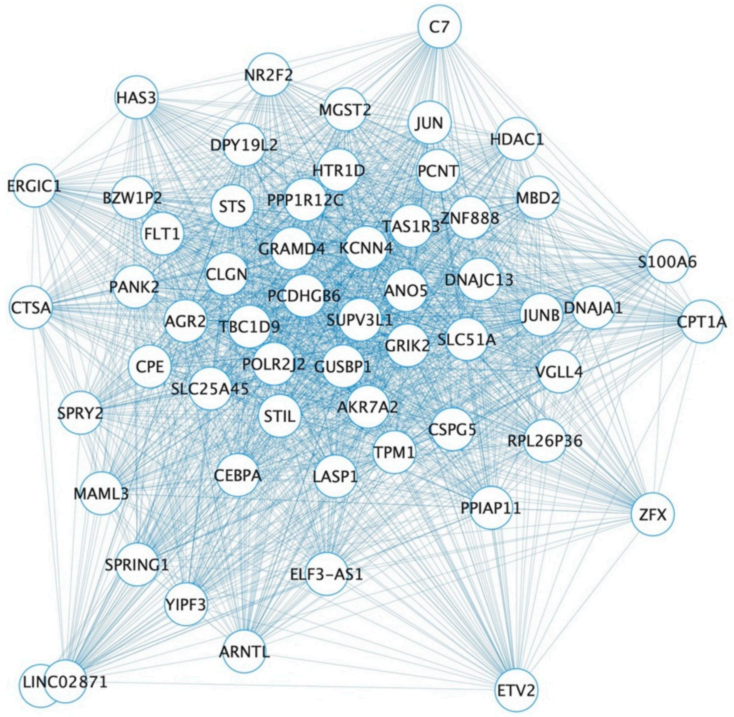

Figure S10: Overview of the lightcoral module. ME expression was increased in females.

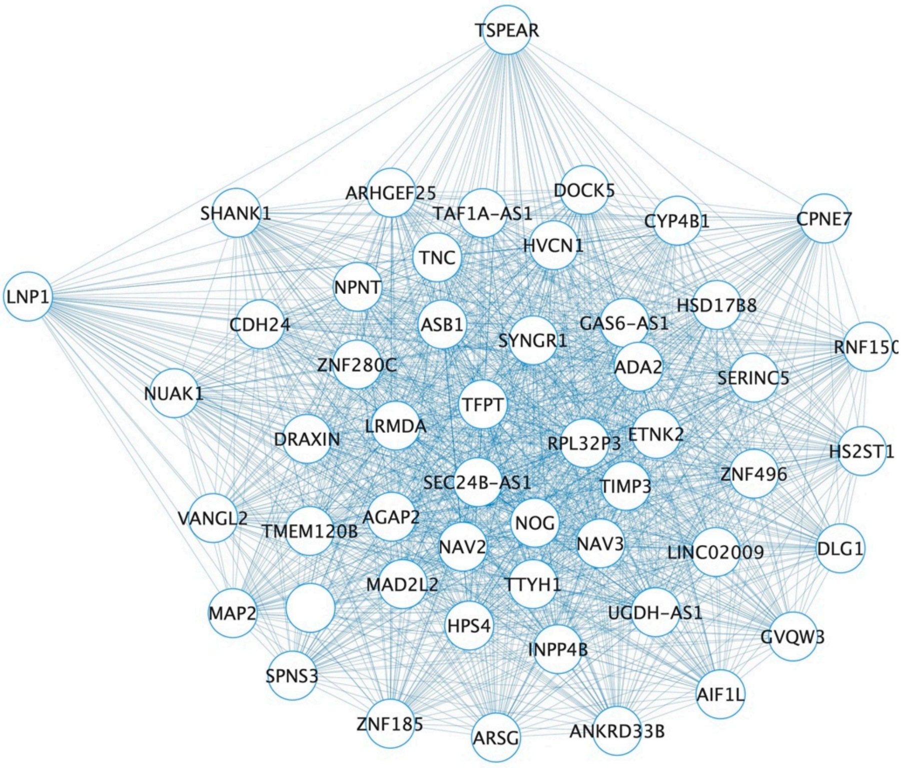

Figure S11: Overview of the plum3 module. ME expression was increased in males.

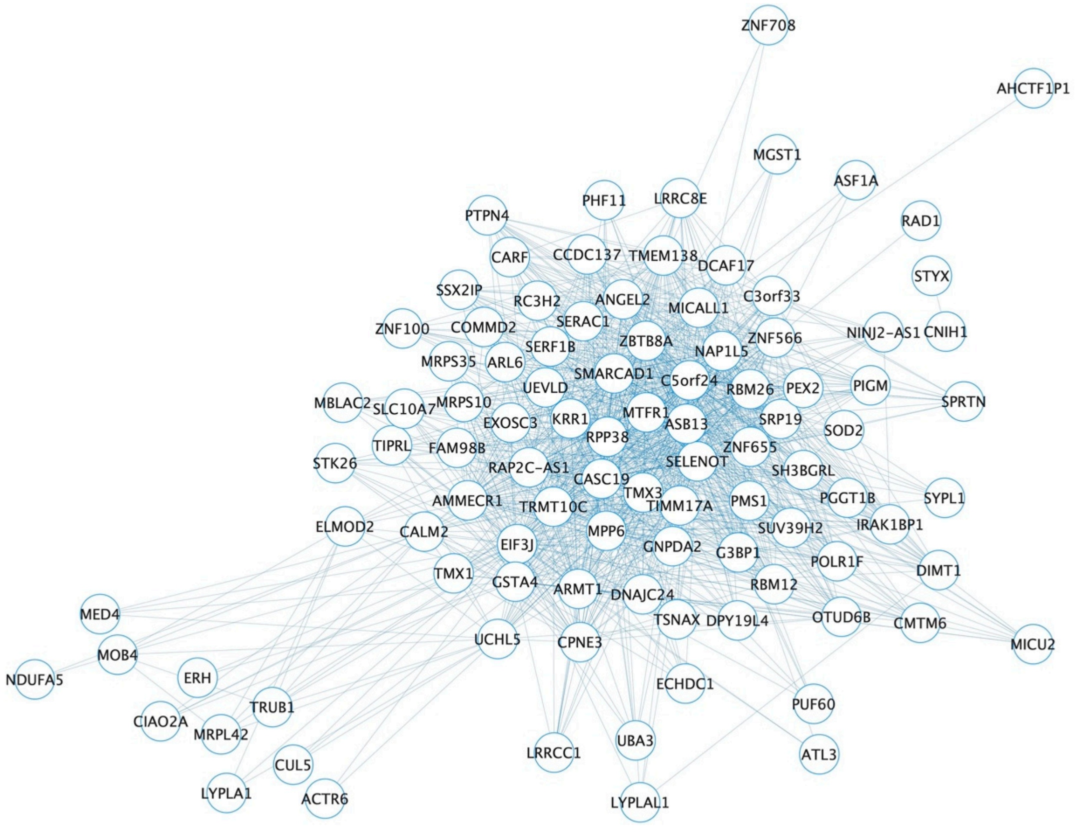

Figure S12: Overview of the lightcyan1 module. ME expression was increased in males. Given the size of the module, a networking threshold of 0.12 was set to reduce poorly weighted connections
